# Direct cost of cochlear implants in Germany – a strategic simulation

**DOI:** 10.1186/s13561-022-00405-8

**Published:** 2022-12-24

**Authors:** Christin Thum, Thomas Lenarz, Steffen Fleßa

**Affiliations:** 1 Department of General Business Administration and Health Care Management, Faculty of Law and Economics, University of Greifswald, Friedrich-Loeffler-Str. 70, 17489 Greifswald, Germany; 2 Department of Otolaryngology, Head & Neck Surgery, Hannover Medical School, Hannover, Germany

**Keywords:** Cochlear implant, Simulation, System dynamics, Health insurance, Cost

## Abstract

**Background:**

Despite the current undersupply of cochlear implants (CIs) with simultaneously increasing indication, CI implantation numbers in Germany still are at a relatively low level.

**Methods:**

As there are hardly any solid forecasts available in the literature, we develop a System Dynamics model that forecasts the number and costs of CI implantations in adults for 40 years from a social health insurance (SHI) perspective.

**Results:**

CI demand will grow marginally by demographic changes causing average annual costs of about 538 million €. Medical-technical progress with following relaxed indication criteria and patients’ increasing willingness for implantation will increase implantation numbers significantly with average annual costs of 765 million €.

**Conclusion:**

CI demand by adults will increase in the future, thus will the costs for CI supply. Continuous research and development in CI technology and supply is crucial to ensure long-term financing of the growing CI demand through cost-reducing innovations.

**Supplementary Information:**

The online version contains supplementary material available at 10.1186/s13561-022-00405-8.

## Introduction

Hearing loss is a widespread health problem. Among adults, age-related hearing loss (presbyacusis) mainly caused by aging processes and noise exposure is most common [1]. Every third person over 65 years develops hearing loss that is to be treated [2]. In cases of severe to profound hearing loss, CI therapy is indicated [3]. Although CI implantation is a well-established standard procedure that is covered by the statutory health insurance (SHI) there is still a major undersupply of CIs in Germany. Only 5–7% of those adults who could benefit from CI are CI users [4]. Currently, about 3,000 to 4,000 new CIs are implanted in Germany every year [5, 6] while according to estimations, approx. 10,000 operations per year would be needed to cover undersupply [7].

In the literature, increasing CI demand is predicted due to increasing demographic ageing, rising prevalence of hearing impairment, growing acceptance of CI therapy and further relaxation of indication criteria [7–9]. However, solid quantitative forecasts of future CI demand and associated costs are hardly available in the literature. Some publications provide rough mathematical approaches or estimates of future CI demand [8, 10]. Neubauer and Gmeiner (2011) predicted increasing services for the hearing-impaired with a maximum being reached in 2040 due to demographic change [11]. Already today, diseases of the ear account for annual costs of 3.2 billion euros (ICD-10 group H60-95) [12]. This paper intents to provide a realistic prognosis that not only calculates CI demand and costs according to hearing loss prevalence, but also considers other crucial variables such as the patients’ attitude towards CIs, since hearing loss is nonlethal and CI implantation is an elective and optional therapy. Through the simulation of likely future scenarios of CI therapy and use, future CI demand and costs can be estimated. The prognosis can serve for planning expenditures and design of standard care for CI therapy in the years to come.

## Methods

We develop a System Dynamics model which is a standard mathematic prognostic approach [13]. The model simulates the number and cost of CI implantations of the adult population for 40 years (2018–2057). We define “adult” as population of 20 years or above, i.e. a simple cohort simulation does not permit to calculate the number of future implantations since a segment of the future adult population has not been born at the beginning of the forecasting process. Consequently, we have to model the entire demographic process with fertility, mortality and aging. Based on the categorization of Kim et al., the model presented here is dynamic, open, deterministic and aggregate [14]. It simulates the demography of Germany, the incidence of severe to profound hearing loss and the implantations of uni- and bilateral CIs. Since the majority of the German population – 88% in 2020 [15] – is insured in the SHI, which is the primary payer of CI care in Germany, the simulation is conducted from the perspective of the SHI.

### Model

**Fig. 1:**
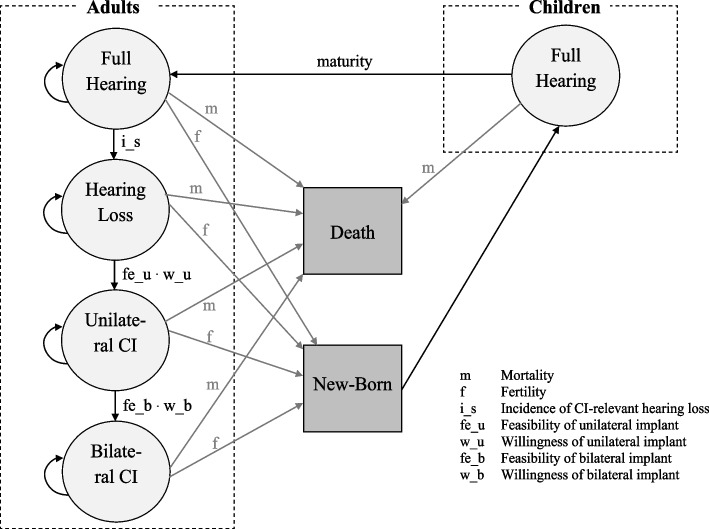
Basic Model (ignoring age-sets)

Figure 1 exhibits the basic structure of the dynamic model. The model ignores hearing loss of children and focusses on age-related adult hearing loss (≥ 20 years), i.e. it is assumed that all adults have full hearing with the age of 20 years. A certain percentage of each age-set develops severe to profound hearing loss (> 60 decibel hearing level **(**dB HL)) which is the indicator condition for CI therapy. The share of the population with CI-relevant hearing loss who fulfils the medical preconditions and is willing to be operated on receives a unilateral implant. Since age-related hearing loss occurs symmetrically, a share of unilaterally implanted persons can receive a second CI for the contralateral ear at a later time, provided – again – that indication criteria are met and willingness for implantation is given (sequential bilateral CI implantation). A CI system consists of the CI implant and the externally worn speech processor, each with different lifespan. In addition, we model the full demographic system with a mortality rate for each age-set, fertility for the years 15 to 49, and aging.

The population is stratified according to age (0..100 years), and disease stage (healthy, CI-relevant hearing loss, unilateral CI, bilateral CI). The following variables are defined:

**Table:** 

*P*_*t,a,h*_	Population in time t in age a in health status h with a = age [years], a = 0..100
	$$h=\left\{\begin{array}{cc}1& healthy\\ 2& hearing loss\\ 3& unilateral CI\\ 4& bilateral CI\end{array}\right.$$
CIu_t,a_	New unilateral CIs in time t in age a; a = 0..100
CIb_t,a_	New bilateral CIs in time t in age a; a = 0..100
SN_t_	New speech processors in time t
CIN_t_	Reimplantations in time t
$\Delta {P\_Fer}_{t,\mathrm{a},\mathrm{h}}$	Change of population in time t in age a in health status h due to fertility, a = 0..100; h = 1..4
$\Delta {P\_Mor}_{t,a,h}$	Change of population in time t in age a in health status h due to mortality, a = 0..100; h = 1..4
$\Delta {P\_Aging}_{t,a,h}$	Change of population in time t in age a in health status h due to aging, a = 0..100; h = 1..4
$\Delta {P\_S}_{t,a,\mathrm{h}}$	Change of population in time t in age a in health status h due to CI-relevant hearing loss, a = 20..100, h = 1..2
$\Delta {P\_CIu}_{t,a,\mathrm{h}}$	Change of population in time t in age a in health status h due to unilateral CI implantation, a = 20..100; h = 2..3
$\Delta {P\_CIb}_{t,a,\mathrm{h}}$	Change of population in time t in age a in health status h due to bilateral CI implantation, a = 20..100; h = 3..4

In addition, we define the following constants (if not stated differently: per year):

**Table:** 

*f*_*t,a*_	Fertility of age a in time t, a = 0..100
*m*_*t,a*_	Mortality of age a in time t, a = 0..100
*i_s*_*a*_	Incidence of CI-relevant hearing loss in age a, a = 0..100
*w_u*_*a*_	Willingness of unilateral implant in age a, a = 0..100
*fe_u*_*a*_	Feasibility of unilateral implant in age a, a = 0..100
*w_b*_*a*_	Willingness of bilateral implant in age a, a = 0..100
*fe_b*_*a*_	Feasibility of bilateral implant in age a, a = 0..100
*fe*_*t*_	Feasibility in year t
*w*_*t*_	Willingness in year t
*l*_*t*_	Lifespan in year t
*s*	Simulation time
*l_s*	Average length of life of speech processor
*l_i*	Average length of life of implant

Based on discrete time steps of one day, the model calculates difference equations (∆P_t,a,h_ = P_t+1,a,h_ – P_t,a,h_) and adjusts the compartments accordingly as it is standard for system dynamic models [16]. As this model attempts a long-term prediction, it is necessary to incorporate a complete demographic model:

#### Demography

##### Fertility

The model calculates the number of births with age-specific fertility rates and adds the newborn to the respective compartment:

$$\Delta{P\_Fer}_{t,0,1}=\sum_{a=15}^{49}\sum_{h=1}^4P_{t,a,h}\cdot f_{t,a}$$

##### General mortality

Each compartment is reduced by mortality:

$$\Delta{P\_Mor}_{t,a,h}=P_{t,a,h}\cdot m_{t,a}\text{ for a=0..100, h=1..4}$$

##### Aging

For every simulation period the respective part of the population is transferred to the higher age-set. An individual reaches the next age set after 365 days:

$$\Delta{P\_Aging}_{t,a,h}=-P_{t,a,h}\,\text{for a=0..100, h=1..4}$$

$$\Delta{P\_Aging}_{t,a+1,h}=P_{t,a,h}\text{ for a=0..99, h=1..4}$$

#### Incidence

The transition between disease stages “healthy” and “hearing loss” is calculated as follows:

$$\Delta{P\_S}_{t,a,1}={-P}_{t,a,1}\cdot{i\_s}_a\text{ for a=20..100}$$

$$\Delta{P\_S}_{t,a,2}=P_{t,a,1}\cdot{i\_s}_a\text{ for a=20..100}$$

#### Implantations

Each simulation period (1 day), the number of new implants, speech processors (external part of the CI system) and reimplantations is calculated and the compartments are adjusted accordingly:

##### New unilateral CIs

$${CIu}_{t,a}=P_{t,a,2}\cdot{fe\_u}_a\cdot{w\_u}_a\text{ for a=20..100}$$

$$\Delta{P\_Clu}_{t,a,2}={-CIu}_{t,a}\text{ for a=20..100}$$

$$\Delta{P\_Clu}_{t,a,3}={CIu}_{t,a}\text{ for a=20..100}$$

##### New bilateral CIs

$${Clb}_{t,a}=P_{t,a,3}\cdot{fe\_b}_a\cdot{w\_b}_a\text{ for a=20..100}$$

$$\Delta{P\_Clb}_{t,a,3}={-CIb}_{t,a}\text{ for a=20..100}$$

$$\Delta{P\_Clb}_{t,a,4}={CIb}_{t,a}\text{ for a=20..100}$$

##### New speech processors

Bilaterally implanted individuals, i.e. the population in time *t* in age *a* in health stage *4* (*P*_*t,a,4*_), receive two new processors:

$${SN}_t=\frac{\sum_{a=20}^{100}P_{t,a,3+}2P_{t,a,4}}{l\_s}\text{ for a=20..100}$$

##### Reimplantations

Bilaterally implanted individuals, i.e. the population in time *t* in age *a* in health stage *4* (*P*_*t,a,4*_), receive two new implants:

$${CIN}_t=\frac{\sum_{a=20}^{100}P_{t,a,3+}2P_{t,a,4}}{l\_i}\text{ for a=20..100}$$

At the end of each simulation period (1 day) the respective compartments are adjusted, i.e.

$${P}_{t+1,a,h}={P}_{t,a,h}+\Delta {P\_Fer}_{t,\mathrm{a},\mathrm{h}}-\Delta {P\_Mor}_{t,a,h}+\Delta {P\_Aging}_{t,a,h}+\Delta {P\_S}_{t,a,\mathrm{h}}+\Delta {P\_CIu}_{t,a,\mathrm{h}}+\Delta {P\_CIb}_{t,a,\mathrm{h}} \text{for a=0..100, h=1..4}$$

The model is implemented in Delphi XE.

Since the analysis targets the expected budget impact of CI implants for the SHI in the coming years, we calculate the total cost per year. Costs are not discounted to a present value, because we assume CI therapy unit costs to remain constant.

### Data

Most parameters of the dynamic model could be taken from the existing literature and national data bases. Table 1 shows the data sources:

**Table 1:** Basic Data of the Standard Simulation

**System**	**Parameter**	**Source**	**Value**
Demography	Population in 2017	[17]	diverse
	Fertility in 2017	[18]	diverse
	Mortality in 2017	[19]	diverse
Hearing Loss	Prevalence	[20]	diverse
	Incidence		adjusted
Implantation	Feasibility of uni- and bilateral CI	Survey	diverse
	Willingness for uni- and bilateral CI	Survey	diverse
	Unilateral CI implantations in 2018	[21]	3,887
	Unilateral CI implantations in 2019	[22]	4,232
	Unilateral CI implantations in 2020		3,809
Technical System	Average length of life of speech processor	[23]	5 yrs
	Average length of life of CI		20 yrs
Cost	Cost of first year of implantation	[23]	33,442.86 €
	Cost of second year after implantation		3,369.96 €
	Cost of all further years p. a		620.73 €
	Cost of reimplantation		27,449.19 €
	Cost of processor exchange		9,847.02 €

We define 2017 as the base year and choose data on demography accordingly. The prevalence rates of hearing loss are only given for classes of 10 years. Consequently, we approximated age-specific rates by estimating an exponential function with ordinary least square. The incidence was taken as the first derivative of the prevalence.

Feasibility and willingness parameters are based on an online survey of 124 German ear nose and throat (ENT) physicians conducted in October 2019. Participants were asked to estimate the medical feasibility of CI implantation (e.g. presence of sensorineural hearing loss, anatomical requirements, positive prognosis of rehabilitation) for potential CI candidates (> 60 dB HL) in different age groups (20–29, …, 80–89, + 90 years) from 0–100%. The doctors also estimated the proportion of patients with medical feasibility who actually receive the treatment in the end, i.e. have willingness for CI implantation (0–100%) for the defined age groups. Feasibility and willingness were surveyed for uni- and bilateral CI therapy, respectively. The questionnaire was pretested by two CI specialists of two different CI centers in Germany.

According to our survey, medical feasibility for CI significantly decreases with age for candidates for both uni- and bilateral implantation (over 80% of 20–29 year-olds vs. under 30% of those over 90 years). Some ENT physicians commented that this can be attributed to more frequent contraindications in higher age groups, such as low rehabilitation prospects, dementia, the presence of other diseases and an increased risk of anesthesia. If the patient already has one CI, the feasibility for a second CI was assessed to be slightly higher than for unilateral implantation in every age group (Table 2).

**Table 2:** Feasibility for CI implantation (survey results)

**Age group**	**Feasibility for CI treatment [%]**				**Age group**	**Feasibility for CI treatment [%]**			
	**Unilateral CI**		**Bilateral CI**			**Unilateral CI**		**Bilateral CI**	
	**mean**	**standard deviation**	**mean**	**standard deviation**		**mean**	**standard deviation**	**mean**	**standard deviation**
20–29	76.57	21.60	82.81	22.94	60–69	58.36	27.40	63.33	33.25
30–39	75.60	19.70	79.74	22.80	70–79	48.54	29.09	50.87	34.61
40–49	71.04	21.95	76.02	24.23	80–89	35.82	30.08	38.04	34.81
50–59	63.80	24.83	69.63	28.35	90 +	27.30	32.31	30.94	36.88

With regard to the patients’ willingness for CI, ENT doctors estimated the majority of younger CI candidates to be willing to get implanted. Among the 20–29 year-olds that would meet the indication criteria, only 31% reject unilateral CI implantation; in the age group of 80–89 years, rejection has doubled (64%). If the patient already has one CI, the willingness for a second CI is slightly higher in younger and slightly lower in older age groups (Table 3).

**Table 3:** Willingness for CI implantation (survey results)

**Age group**	**Willigness for CI treatment [%]**				**Age group**	**Willingness for CI treatment [%]**			
	**Unilateral CI**		**Bilateral CI**			**Unilateral CI**		**Bilateral CI**	
	**mean**	**standard deviation**	**mean**	**standard deviation**		**mean**	**standard deviation**	**mean**	**standard deviation**
20–29	68.64	23.68	72.92	27.72	60–69	42.70	23.29	46.62	30.14
30–39	67.82	22.39	70.70	27.14	70–79	30.94	22.83	24.70	21.48
40–49	60.38	23.67	65.97	28.64	80–89	19.76	20.92	17.08	22.20
50–59	53.36	23.46	60.70	29.84	90 +	13.12	21.08	10.32	21.23

### Model verification and validation

**Fig. 2:**
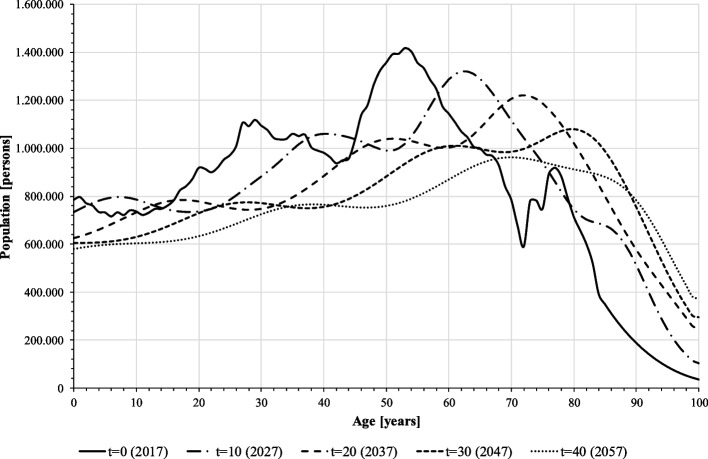
Demographic prognosis: population composition (source: own simulation)

The model is tested for correct implementation of the prognosis calculations. The simulation shows long-term trends in population shrinkage and ageing (Fig. 2), which is in line with current population projections [24], so the demographic system can be assumed to be verified and validated. However, the forecasted number of more than 50,000 new CIs p.a. is extremely high and does not represent the current situation in Germany. The input data of the model are therefore checked again for plausibility. While prevalence and incidence of CI-relevant hearing loss were extracted from epidemiological studies and are likely to have only minor biases, self-collected data on feasibility and willingness for CI may contain uncertainties. In particular, patient willingness as the ultimate reason for CI implantation must have been overestimated by the ENT doctors surveyed: The self-collected data on CI willingness refers to patients who have been recommended a CI and meet the medical criteria for implantation. In the test run, however, this data was applied to the entire population with severe to profound hearing loss, including those who are not suitable for CI (e.g. no recommendation, no sufficient suffering pressure, no reimbursement). To reflect the actual state, willingness is therefore calibrated to a very low value of 0.01 corresponding to current implantation numbers for all age groups. CI willingness of 1% – better be seen as the actual CI implantation rate – is used for the simulation of the baseline scenario. With 3,000 to 4,000 simulated annual implantations the simulation represents the status quo of CI demand in Germany [5, 6], so the model and data are assumed to be verified and validated. Data on CI willingness according to our ENT survey is used for scenario simulation instead (Scenario simulation section).

### Scenarios

In order to assess future developments of CI demand and cost, different scenarios are simulated (3.3). For the scenario simulation of technical progress in CI technology according to current research projects (Scenario innovative CI section) extra data is necessary. In a survey of six German interdisciplinary CI experts (e.g. implanter, producer, pharmacist) conducted in February 2020, lifespan and costs of the innovative CI were estimated (see online appendix A1). The data displayed in table 4 resulted and was used for the scenario simulation:

In addition to scenario simulations with simple parameter changes, we test the consequences of steady increases of some parameters over the entire forecast period (4.2), i.e. CI feasibility, willingness and the CI’s lifespan. We assumed a linear increase for each parameter resulting in the following equations:

Increase of CI feasibility:

$${fe}_{t}={fe}_{0}\left(1+\frac{{fe}_{s}-{fe}_{0}}{s}\cdot t\right)$$

Increase of CI willingness:

$${w}_{t}={w}_{0}\left(1+\frac{{w}_{s}-{w}_{0}}{s}\cdot t\right)$$

Increase of device lifespan:

$${l}_{t}={l}_{0}\left(1+\frac{{l}_{s}-{l}_{0}}{s}\cdot t\right)$$

## Results

### Baseline scenario

If willingness for implantation (resp. CI implantation rate) remains at the current low level (0.01), implantation numbers in Germany will increase only minimally over the next 40 years. 

**Fig. 3:**
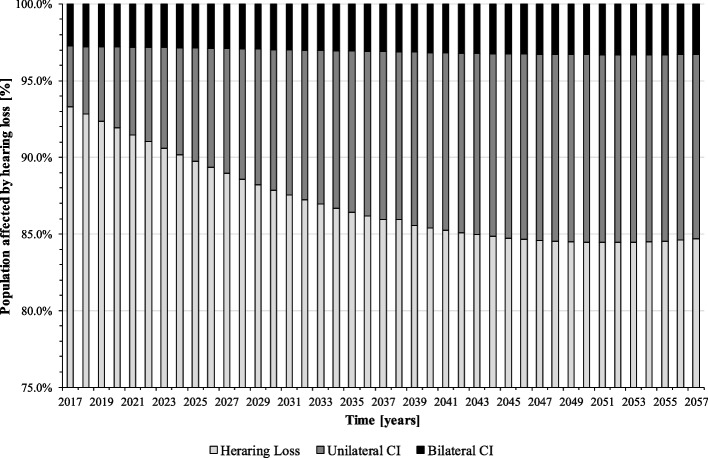
Population by disease stage, ignoring the “healthy” (baseline scenario) (source: own simulation)

Figure 3 shows the extensive undersupply of CI in the population. The majority of CI-relevant hearing impaired people does not receive an implant and remains in the health stage “hearing loss”. In the base year 2017, the shares of unilateral and bilateral CI users in the population affected by hearing loss are only at 4% (24,944 persons) and 3% (17,501 persons), respectively. Due to demographic change, however, the proportion of older people who are more affected by hearing loss will increase in the following years. As a result, with given CI feasibility and willingness, the proportion of CI users will also increase. In 2057, the model forecasts some 12% (79,302 persons) of the population affected by hearing loss to be unilaterally and 3% (21,804 persons) to be bilaterally implanted. Still, about 85% will be affected by severe to profound hearing loss (751,292 persons).

**Fig. 4:**
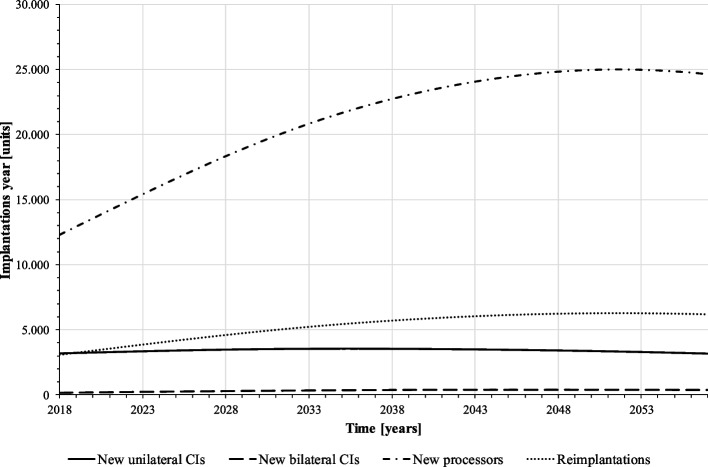
Implantations per year (baseline scenario) (source: own simulation)

Figure 4 exhibits the predicted demand of CI operations per year. On average, about 3,400 unilateral and 330 bilateral implantations are performed per year. In the first simulated years, implantations increase as the baby boom generations move into older age groups with higher prevalence of hearing loss. At the end of the forecast horizon, implantation numbers decrease slightly due to population decline and saturation of the population with CIs. Overall, however, the implantation numbers remain at a rather stable level. The numbers of reimplantations and speech processor exchanges, on the other hand, increase noticeably over the forecast period as there are more implanted CI users among the population over time who potentially need these device changes. Overall, the number of reimplantations roughly doubles over the forecast period. Since processor replacements occur on average every five years, i.e. much more frequently than implantations, their number is already relatively high in the first simulated years.

**Fig. 5:**
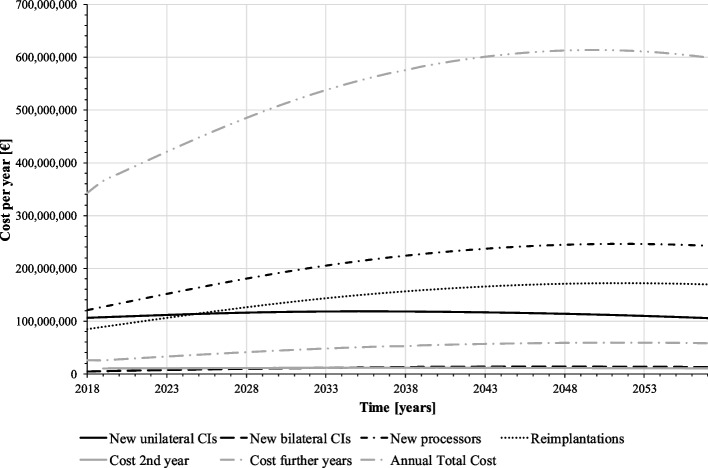
Cost of CI supply per year (baseline scenario) (source: own simulation)

The expected cost for the SHI is based on the forecasted demand for CI. As the number of implanted patients increases over the forecast period, so do the total costs of CI supply (Fig. 5, upper graph). On average, annual costs amount to about 538 million €. The total CI supply cost is composed of different cost components: due to their relatively short lifespan with relatively high unit cost (9,847 € see Table 1), the cost of speech processor replacements make up the largest part of the total cost. The second largest is the cost of unilateral initial implantation, which, however, will be exceeded by the cost of reimplantations from 2025 onwards as the proportion of implanted persons in the population increases. The follow-up costs from the third year after implantation is the fourth largest cost component; even though these are relatively low at 620.73 €, they are incurred per patient until death and can accumulate to a high amount depending on the remaining lifetime. The cost of the second year after implantation comprises only one year of care in a CI patient’s life and the cost for bilateral first implantations occurs relatively rarely, so these cost components are the lowest from the SHI perspective. According to the standard simulation, SHI has to expect a total cost of about 21.5 billion € for CI care in the forecast period (2018–2057).

### Sensitivity

**Fig. 6:**
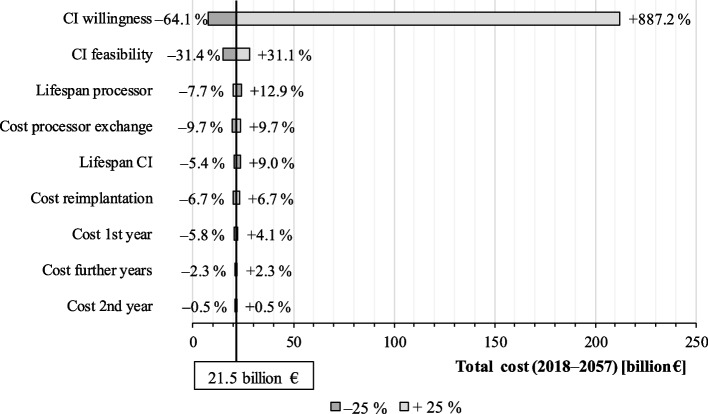
Sensitivity tornado diagram (source: own simulation)

A sensitivity analysis is conducted to determine the impact of each input variable on the overall cost. For this purpose, the input data for CI feasibility, CI willingness and all cost components are increased and reduced by 25% in individual simulations. The total costs of each simulation is compared with the results of the standard simulation (21.5 billion €). As can be seen in Fig. 6, patients’ willingness for CI implantation has the strongest impact on the overall cost for CI care because it is crucial for CI demand. CI feasibility shows the second biggest impact. Cost variables are much less sensitive.

### Scenario simulation

In addition to the standard simulation, three future scenarios are simulated:

#### Scenario higher willingness for CI

This scenario assumes increasing willingness for CI therapy among the German population which already shows in increasing coding of the respective Diagnosis Related Groups (DRG) for CI implantation (D01A, D01B) [6]. Instead of the very low estimated willingness in the baseline scenario, future CI demand and associated cost are calculated with the data on patients’ CI willingness surveyed.

As sensitivity analysis has shown (Fig. 6), higher willingness has a big impact on implantation numbers and will lead to significantly higher cost. In the scenario simulation, implantations increase massively in the first simulated years because the applied higher willingness parameters create a backlog demand which can be seen as the settling phase of the simulation. Over the rest of the forecast period, implantation numbers level on a relatively high level. The population share in the stages "unilateral CI" and "bilateral CI" is increasing accordingly. In 2057, the proportion of CI users (uni- and bilateral) is at 0.84%. In contrast to the baseline scenario, CI undersupply is significantly reduced (see online appendix A2 Population by disease stage, ignoring the “healthy” (scenario higher willingness for CI)).

In total, 1,229,425 new unilateral and 873,229 new bilateral CIs will be implanted in this scenario, which is about 9 and 65 times higher than in the baseline scenario. On average, 30,736 unilateral and 21,831 bilateral first implantations are performed annually. Due to the high number of implantations and CI users, the annual number of reimplantations and speech processor exchanges is also significantly higher than in the baseline scenario. A total of 2,034,562 reimplantations and 8,138,249 processor changes are performed, i.e. almost 10 times as many as in the baseline scenario (210,940 and 843,760). On average, there are 50,864 reimplantations and 203,456 speech processor exchanges per year (see online appendix A3 Implantations per year (scenario higher willingness for CI)).

The very high number of implantations and device changes leads to considerably higher costs. On average, 5.6 billion € are incurred annually for CI care. The largest cost components – again – are the cost for speech processor changes, reimplantations and unilateral implantations. Overall, the cost for SHI is 10 times higher than in the baseline scenario and amount to 222 billion € (2018–2057) (see online appendix A4 Cost of CI supply per year (scenario higher willingness for CI)).

#### Scenario relaxation of indication criteria

CI indication criteria have continuously broadened with technical, surgical and software improvements in CI systems over time. While at the beginning of standard clinical CI therapy only completely deaf patients were implanted [25], a CI is now indicated for patients with severe to profound sensorineural hearing loss (> 60 dB HL), or whenever better hearing can be expected with CI and the possibilities of hearing aids have been exhausted [3, 26]. Today, even patients with moderate hearing loss (> 40 dB HL) and insufficient speech understanding are implanted, with increasing tendency [27, 28], so that a general recommendation of these patient group for CI therapy can be expected in the future.

To simulate this scenario, CI-relevant hearing loss is extended to moderate hearing loss (> 40 dB HL). This leads to a larger stock of potential CI candidates (disease stage “hearing loss”, see online appendix A5 Population by disease stage, ignoring the “healthy” (scenario relaxation of indication criteria)) and results in higher implantation numbers and cost (see online appendix A6 Implantations per year (scenario relaxation of indication criteria) and A7 Cost of CI supply per year (scenario relaxation of indication criteria)). On average, unilateral implantation numbers are five times as high as in the baseline scenario (approx. 16.000 p. a.). Bilateral implantations are only 3 times as high (1.070 p. a.) because they only occur, if the patient is already implanted unilaterally. Additionally, willingness is still as low as in the baseline scenario, so bilateral implantation is less likely. Processor exchanges and reimplantations more than double compared to the baseline scenario.

#### Scenario innovative CI

CI technology has constantly been further developed and improved in performance, efficacy and efficiency [25]. Current research and development projects are working on innovative, anti-inflammatory and anti-proliferative CI coatings to reduce medical induced complications and reimplantations in CI therapy. Therefore, such innovative CIs are expected to have a longer lifespan, less follow-up costs after implantation, but higher CI unit cost due to additional production processes and pharmaceuticals [29, 30].

To simulate this scenario, we used the data collected in our survey of CI experts (see 2.4, Table 4). Since CI cost data were changed here only, the number of implantations (unilateral and bilateral) and processor exchanges remain at exact same level as in the baseline scenario. The increased lifespan of the implant, however, lowers the reimplantation rate: Compared to the baseline scenario, 27,514 CI reimplantations are prevented in the forecast period (–13%). On average, 688 reimplantations will be saved p. a. (see online appendix A8 Implantations per year (scenario innovative CI)). Still, the cost of reimplantations for the SHI is 0.7% higher in this scenario due to higher implant unit cost. In contrast, the cost in the second year after implantation could be reduced by 1.8% and costs from the third year after implantation are also 3.0% lower when using the innovative CI. However, since these cost components only have slight influence on the overall cost (see Fig. 6), this improvement is of little significance. In total, the cost of CI supply using the innovative implant amount to 22.1 billion € (2018–2057), which corresponds to a total cost increase of 2.8% compared to the baseline scenario (see online appendix A9 Cost of CI supply per year (scenario innovative CI)).

**Table 4:** Scenario input data innovative CI

**System**	**Parameter**	**Source**	**Value**
Technical System	Average length of life of CI	Survey	23 yrs
Cost	cost of first year of implantation		37,578.71 €
	cost of second year after implantation		3,310.69 €
	cost of all further years p. a		602.21 €
	cost of reimplantation		31,777.68 €

## Discussion

### Scenario evaluation

The simulation results indicated an increase in CI demand and cost for each scenario investigated. The increase is based on different factors in each scenario:

#### Baseline scenario

Here, the increase in CI demand is due to the rising proportion of older people, who have a higher incidence of hearing loss, leading to higher implantation numbers. Thus, demographic change will ensure a natural expansion of CI demand in Germany, even if all other variables such as CI indication criteria, individual CI feasibility or patients’ willingness to undergo implantation remain unchanged. However, the results also show a saturation of the population with new CIs, especially after the supply and death of the baby boom generations, and declining CI demand due to population decline in the second half of the forecast period. In all, the baseline scenario shows the inevitable minimum expansion of future CI demand resulting from future demographic developments.

#### Scenario higher willingness for CI

The original intention of the survey of ENT physicians on CI willingness was not to determine the future, but the status quo. However, the survey results led to unrealistically high implantation numbers for baseline simulation in our prognosis model. Apparently, the ENT physicians surveyed already perceive a high CI willingness in the population today, so the future scenario of increased willingness seems very likely. On the other hand, the survey results are not representative, as the sample is relatively small and findings may be distorted, e.g. by selection bias. Furthermore, the determination of CI willingness as a single model variable is difficult because in reality, it depends on several individual decisions and needs of the patient. For example, cost coverage by the SHI is a central factor [31]. Another important determinant could be the individual level of speech recognition ability respectively the degree of suffering from hearing loss, which can vary widely among CI candidates [32].

In any case, such a massive and abrupt increase in CI willingness as assumed in this scenario seems unrealistic. Future CI demand is therefore overestimated in this scenario. A slowly increasing willingness for CI in the population can rather be assumed. Here, other trends such as increasing use of technology [33], generally rising demands for quality of life and increasing health awareness [34] probably also play a role.

#### Scenario relaxation of indication criteria

The increasing number of CI candidates with only moderate hearing loss suggests a further future expansion of the indication in favor of this patient group. This would result in considerably more CI implantations than in the status quo (see online appendix A6). However, the prognosis model calculates with full implementation of the new indication from 2018, although in fact, it is difficult to estimate when exactly a general indication expansion could be recommended in the CI care guidelines. Thus, CI demand is overestimated in this scenario as well. In the recently published new version of the German CI care guideline (October 2020 [3]), the target group for implantation is still characterized as having at least severe hearing loss (> 60 dB HL). The guideline is valid for five years, so no official change in indication is expected for the time being. However, further health care research on the benefits of CI therapy in this patient group is necessary first.

Furthermore, the scenario overestimates the extent of the implementation of the indication relaxation in health care practice. The model assumes an assured CI candidacy for individuals in the stage of CI-relevant "hearing loss". In fact, a large share of patients with moderate hearing loss will still be treated well with hearing aids. Besides, CI candidacy also depends on the attitudes of the ENT physicians: if they are not convinced of CI treatment for the moderately hearing impaired, they will not recommend it to them. However, the doctor’s advice is essential for the patient to even consider CI implantation.

Overall, the scenario of indication expansion can be assessed as a likely future development. However, the timing and the extent of its implementation in health care practice are uncertain.

#### Scenario innovative CI

Analogous to the scenarios of relaxed indication criteria and increased CI willingness, this scenario assumes the widespread use of the innovative CI from 2018 and therefore overestimates its consequences for overall CI cost. Until now, however, it is still unclear whether and when the new CI will be available in standard care and to what extent it will be used by the implanting hospitals. The probability of this scenario occurring depends on the success of the innovation process of the new implant.

### CI demand and cost prognosis

The scenarios examined are all likely to occur in the future, so their respective effects will mix in reality. However, the results show that immediate increases in CI feasibility (respectively a broader indication for CI), CI willingness an CI performance (respectively a longer lifespan) are unrealistic. Medical technological progress and patients’ growing acceptance for CI therapy can instead be seen as a linear increase.

Therefore, we create a mixed scenario combining the most likely events derived from our considerations in Scenario evaluation section with sharpened assumptions, i.e. linear increase of the data. For this mixed prognosis scenario we assume the following:

- CI feasibility will double over the forecast period (+ 100%) because continuous progress in CI technology, insertion technique, anesthesia and rehabilitation as well as gradual relaxation of indication criteria (e.g. > 40 dB HL) will allow a broader spectrum of patients to benefit from CI implantation.
- Technological and medical progress will increase the longevity of CI systems over the next 40 years. We assume the implant to reach a 30-year lifespan and the speech processor to reach 10 years of service life until 2057.
- CI willingness will increase due to higher acceptance of this therapy. We assume an increase from 0.01 in 2017 to the significantly higher parameters according to ENT physicians’ expectations (Table 3) in 2057.

The linear increases of the variables were programmed as described in Scenarios section. As both cost increase through innovation and cost decrease through economies of scale are possible in the future, cost parameters remain unchanged in the mixed prognosis scenario.

**Fig. 7:**
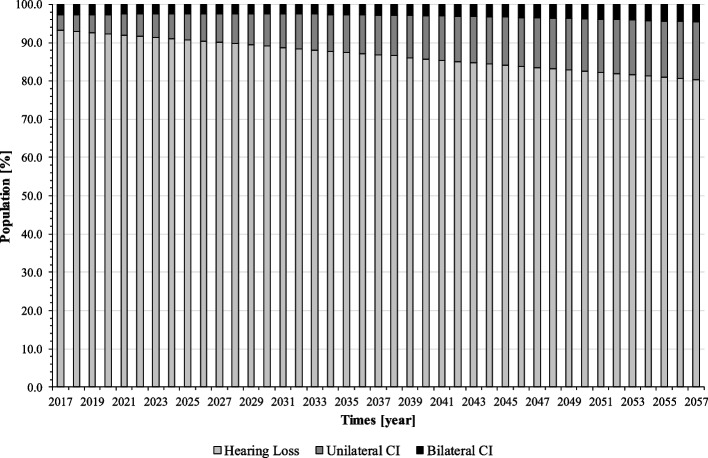
Population by disease stage, ignoring the “healthy” (mixed prognosis scenario) (source: own simulation)

**Fig. 8:**
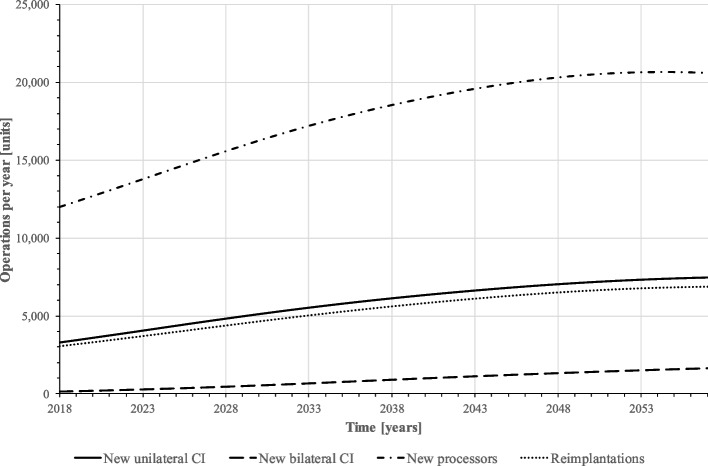
Implantations per year (mixed prognosis scenario) (source: own simulation)

**Fig. 9:**
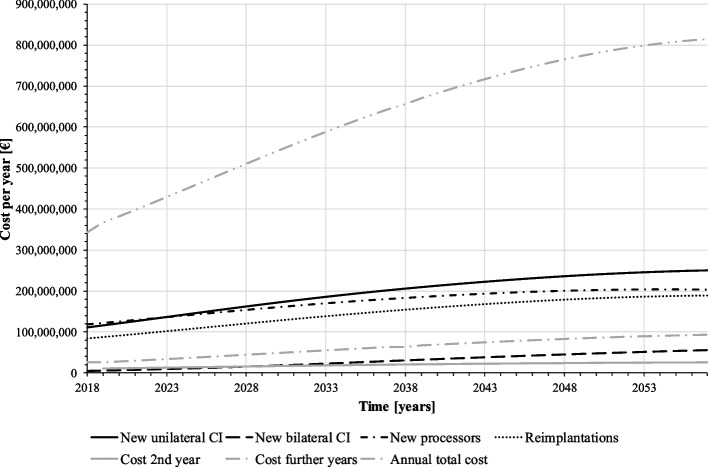
Cost of CI supply per year (mixed prognosis scenario) (source: own simulation)

Figures 7, 8 and 9 present the results: according to our simulation, the number of uni- and bilateral implantations per year will increase from 3,441 in 2018 to 9,107 in 2057 (+ 265%). In the second half of the forecast period, numbers grow with a lower rate. On average, 5,820 unilateral and 879 bilateral CI implantations are performed on adults annually. Reimplantations and processor upgrades increase by 226% and 172%, respectively (Fig. 8). However, increasing CI demand cannot compensate CI undersupply in the forecast period, the population in health stage “hearing loss” remains at a high level (Fig. 7). Total annual cost for CI care grows from 343 million € in 2017 to 814 million € in 2057. Compared to the baseline scenario, the average annual cost for CI supply will increase by 16% (538 million € vs. 626 million € p.a.).

Testing the new assumptions in individual simulations reveals the significance of the CI’s lifespan for expenditure planning for the SHI (Table 5): Compared to the baseline scenario, a higher lifespan of devices considerably reduces annual CI supply cost by 19% (“lifespan increase”). If increasing CI feasibility and willingness are assumed, it generates cost reductions of 18% (“mixed prognosis scenario”).

**Table 5:** Cost forecast simulations (source: own simulation)

**Scenario**	**CI Feasibility**	**CI willingness**	**Device lifespan**	Ø **Cost per year [million €]**
Baseline	no change	no change	no change	538
Lifespan increase	no change	no change	in t = 40 *l_i* = 30, *l_s* = 10	437
Feasibility and willingness increase	in t = 40 + 100%	in t = 40 acc. to survey	no change	765
Mixed prognosis scenario	in t = 40 + 100%	in t = 40 acc. to survey	in t = 40 *l_i* = 30, *l_s* = 10	626

Technical progress, on the other hand, may not only result in higher lifespans of devices and cost reductions, but also causes the need for early device exchanges, e.g. if older CI systems no longer allow maintenance and upgradability, or if better hearing results can be expected with newer implants. In the long term, however, growing CI demand holds the potential for economies of scale in CI production which can have particular cost-saving effects, since CI unit cost accounts for the majority of CI therapy cost. Simplifications and innovations in the treatment process such as innovative remote care, telemedicine and self-fitting programs offer further potential for future cost reductions [35].

### Uncertainty

The analysis presented here clearly shows that strategic modelling in healthcare is challenged by a high degree of uncertainty. We face a dilemma: on the one hand side, we have to forecast the numbers of interventions and their long-term budget impact [36]. On the other hand, the longer the time horizon the higher the degree of uncertainty [37]. Firstly, there is uncertainty considering medical facts. Although much is known about the medical dimension of CIs, there is still a risk that future generations will know much more and may, for instance, have a pharmaceutical remedy against hearing loss. This leads, secondly, to an uncertainty of medical care structures and data. For instance, predicting the unit cost of hearing loss is only feasible if the clinical pathway and the prevalence rates are known. At the same time, our analysis focusses on patients of the statutory health insurance. We cannot assess whether privately insured will behave differently. Besides, we collected data in own surveys that are likely to contain considerable uncertainties. For instance, this shows in the high standard deviations of data on CI feasibility and willingness (Tables 2 and 3), which simultaneously have the biggest impact on implantation numbers and cost in our forecast model (Fig. 6).

Thirdly, many economic facts are unknown. For instance, we calculated the expenditure for the statutory health insurance (which are known), but other costs (e.g. household cost, providers’ costs) are unknown (at least in Germany).

Consequently, all results of this model are under extreme uncertainty and must be handled with great caution. The figures suggest a degree of precision which no simulation can ever offer. However, there is still a lot to learn from these strategic models as they are “modelling for insights, not for numbers” [38]. The health economic model is designed to understand the system much better, to point at research gaps and to give some principle answers to pressuring questions, such as the budget impact under current assumptions. These answers will not be precise figures, but still allow policy advice. Our simulations clearly indicate a strong increase of cost for the health insurers, and this is a robust insight which does not depend on the uncertainty of structures and parameters.

## Conclusions

CI demand by adults in Germany and the related cost for CI supply for the SHI are going to increase in the future. Demand will grow due to demographic aging and its related changes in the spectrum of disease towards chronic diseases such as hearing loss. Additionally, technical progress leading to an expanded CI indication and increasing acceptance of CI therapy among hearing impaired people will raise implantation numbers and reduce the current undersupply with CI among potential candidates. CI implantation will become a common treatment. This can be seen as a favorable development since hearing loss is a risk factor for various other care- and cost-intensive diseases (e.g. dementia, depression, fractures) [39–41] that can be prevented. Compared to the status quo, we expect annual CI supply cost to rise by 16% over the next 40 years, whereby gradual treatment process optimization and economies of scale also may unfold cost-saving effects.

In order to secure CI treatment for all potential CI candidates in the future, further research and improvement of CI technology and supply is crucial to achieve long-term cost reductions. Innovations that focus on the longevity of the CI system are particularly promising.

However, the high costs clearly show that more efforts and strategies are needed to finance health care treatments of the elderly. Prevention of noise-induced hearing loss and health education programs could be useful and probably also be cost-effective.

## Supplementary Information

**Additional file 1.** 2-Stage Delphi Survey of CI experts in Germany: Results Stage 2.

**Additional file 2. **Population by disease stage, ignoring the “healthy” (scenario higher willingness for CI).

**Additional file 3. **Implantations per year (scenario higher willingness for CI).

**Additional file 4. **Cost of CI supply per year (scenario higher willingness for CI).

**Additional file 5. **Population by disease stage, ignoring the “healthy” (scenario relaxation of indication criteria).

**Additional file 6. **Implantations per year (scenario relaxation of indication criteria).

**Additional file 7. **Cost of CI supply per year (scenario relaxation of indication criteria).

**Additional file 8. **Implantations per year (scenario innovative CI).

**Additional file 9. **Cost of CI supply per year (scenario innovative CI).

## Data Availability

Some of the datasets used are publicly available (e.g. population data, see references). Other datasets used were determined in own surveys. Own data and data generated in simulations and analysed during the current study are available from the corresponding author on reasonable request.

## Abbreviations

CI: Cochlear implant
dB HL: Decibel hearing level
SHI: Statutory Health Insurance
