# Supplementary material for: Direct cost of cochlear implants in Germany – a strategic simulation
Source: Health Econ Rev. 2022 Dec 24;12:64. doi: 10.1186/s13561-022-00405-8 (PMC9789618; doi:10.1186/s13561-022-00405-8)
Supplement: Supplementary file 1 — Additional file 1. 2-Stage Delphi Survey of CI experts in Germany: Results Stage 2. [file 13561_2022_405_MOESM1_ESM.docx]

2-Stage Delphi Survey of CI experts in Germany: Results Stage 2

**How will the costs of CI therapy change if CIs with innovative coating are used?**

- Survey period: December 2019 – February 2020
- Number of participants: 6
- Professions of participants: ENT doctor/CI implanter, pharmacist, CI manufacturer, economist
- Number of answers to individual questions varies (see below)

| **Differences between the innovative and the established CI system** |
| --- |

***Components of the established CI system:*** *microphone, speech processor with transmitter coil, battery, receiver coil, electrode cable, electrodes.*

**CI components** (n=6)

- 100 % of the survey participants stated that the innovative CI system will consist of the same components and will not differ from the CI system already established on the market in this regard.

**Materials and quantities** (n=6)

- 100 % of participants stated that the additional and innovative material component ist he CI coating that contains a pharmaceutical active substance (pharmaceutical active substance + carrier polymer), whereby the polymer and active substance are still to be finally determined.
- The coating is applied to the carrier material of the electrode and/or the receiver coil, housing and cable, which is also still to be finally decided (100 % of the answers).
- Additional material quantities in the micro- to milligram range are required for each innovative implant (100 % of responses).

**Manufacturing processes** (n=6)

- 100 % of the survey participants indicated that the manufacturing processes of the innovative CI will need to be expanded to include additional coating and possibly microstructuring processes of the carrier material.
- Whether microstructuring will be necessary is yet to be determined. In this case, manufacturers will have to purchase a microstructured injection mould specially developed for this purpose (100 % of responses).
- Furthermore, depending on the carrier polymer that is still to be determined, material mixing processes may have to be changed by manufacturers (100 % of responses).

| **Manufacturing costs of the innovative CI system** |
| --- |

(n=6)

- 100 % of the survey participants assume higher manufacturing costs of the innovative CI compared to the current CI system.
  - 17 % estimate a 10 % cost increase.
  - 50 % estimate a 20 % cost increase.
  - 17 % estimate a 10–50 % cost increase.
  - 17 % do not specify the amount of the cost increase.
- On average, a **20 % increase in manufacturing costs** is estimated.

| **Implantation costs of the innovative CI system** |
| --- |

(n=6)

There is no clear tendency among the participants:

- 50 % assume that **implantation costs will remain the same**.
- 50 % assume **higher implantation costs** for the innovative CI compared to the current CI system.
  - 2 participants estimate a 10 % increase in costs.
  - 1 participant does not specify the amount of the cost increase.

| **Lifespan of the innovative CI** |
| --- |

***Lifespan of the established CI:***

| Median | 20 years* |
| --- | --- |
| Maximum | 40 years** |

*Deutsches Hörzentrum Hannover

**Estimation on the the basis of Deutsches Hörzentrum Hannover

- The majority of participants assume **an increasing average** and an **unchanged maximum** **lifespan** of the innovative CI compared to the currently used CI.

|  | unchanged | increases |
| --- | --- | --- |
| average lifespan (n=6) | 33 % | 67 %; increase 5–25 % (Ø 15 %) |
| maximum lifespan (n=6) | 67 % | 33 %; increase 5–20 % (Ø 12.5 %) |

| **Cost savings through innovative CI after implantation** |
| --- |

***Follow-up costs of the established CI system:***

| **Outpatient personnel costs**  (doctors, audiologists, pedagogues, engineers) | **current** |
| --- | --- |
| 1st year after implantation | 740.92 €* |
| 2nd year after implantation | 370.46 €* |
| from 3rd year after implantation | 185.23 €* |

*Deutsches Hörzentrum Hannover

(n=5)

- 100 % of participants expect cost savings from the innovative CI in the years following implantation.

| Cost savings in the 1st year after implantation | 10–50 % (Ø 26 %) |
| --- | --- |
| Cost savings in the 2nd year after implantation | 5–25 % (Ø 16 %) |
| Cost savings in the 3rd year after implantation | 5–20 % (Ø 10 %) |

| **Notes and Comments** |
| --- |

| – |
| --- |
