# Supplementary material for: Direct cost of cochlear implants in Germany – a strategic simulation
Source: Health Econ Rev. 2022 Dec 24;12:64. doi: 10.1186/s13561-022-00405-8 (PMC9789618; doi:10.1186/s13561-022-00405-8)
Supplement: Supplementary file 3 — Additional file 3. Implantations per year (scenario higher willingness for CI). [file 13561_2022_405_MOESM3_ESM.docx]

Implantations per year (scenario higher willingness for CI).
